# WSB-1 regulates the metastatic potential of hormone receptor negative breast cancer

**DOI:** 10.1038/s41416-018-0056-3

**Published:** 2018-03-15

**Authors:** Flore-Anne Poujade, Aarren Mannion, Nicholas Brittain, Andrew Theodosi, Ellie Beeby, Katarzyna B. Leszczynska, Ester M. Hammond, John Greenman, Christopher Cawthorne, Isabel M. Pires

**Affiliations:** 1 School of Life Sciences, University of Hull, Hull, UK; 2 Cancer Research UK and Medical Research Council Oxford Institute for Radiation Oncology, Department of Oncology, The University of Oxford, Oxford, UK; 3 Positron Emission Tomography Research Centre, University of Hull, Hull, UK

**Keywords:** Breast cancer, Oncology

## Abstract

**Background:**

Metastatic spread is responsible for the majority of cancer-associated deaths. The tumour microenvironment, including hypoxia, is a major driver of metastasis. The aim of this study was to investigate the role of the E3 ligase WSB-1 in breast cancer biology in the context of the hypoxic tumour microenvironment, particularly regarding metastatic spread.

**Methods:**

In this study, WSB-1 expression was evaluated in breast cancer cell lines and patient samples. In silico analyses were used to determine the impact of WSB-1 expression on distant metastasis-free survival (DMFS) in patients, and correlation between *WSB1* expression and hypoxia gene expression signatures. The role of WSB-1 on metastasis promotion was evaluated in vitro and in vivo.

**Results:**

High *WSB1* expression was associated with decreased DMFS in ER-breast cancer and PR-breast cancer patients. Surprisingly, *WSB1* expression was not positively correlated with known hypoxic gene expression signatures in patient samples. Our study is the first to show that WSB-1 knockdown led to decreased metastatic potential in breast cancer hormone receptor-negative models in vitr*o* and in vivo. WSB-1 knockdown was associated with decreased metalloproteinase (MMP) activity, vascular endothelial growth factor (VEGF) secretion, and angiogenic potential.

**Conclusions:**

Our data suggests that WSB-1 may be an important regulator of aggressive metastatic disease in hormone receptor-negative breast cancer. WSB-1 could therefore represent a novel regulator and therapeutic target for secondary breast cancer in these patients.

## Introduction

Breast cancer is the second most common cancer type worldwide, affecting one in eight women in the UK.^1^ Hormone receptor (HR) status is critical in evaluating survival rates and determining therapeutic approaches. Triple negative breast cancer (TNBC) (no detectable oestrogen receptor (ER), progesterone receptor (PR) or human epidermal growth factor receptor (HER2) expression), is associated with both highest metastatic potential and worst overall survival (OS).^2^

Metastatic spread is responsible for the majority of cancer-associated deaths, and it has been extensively demonstrated that tumour microenvironmental factors, such as hypoxia are major drivers of metastatic disease.^3^ Tumour hypoxia arises as a consequence of irregular perfusion of the tumour mass, increased metabolic demand from rapidly proliferating cells, as well as deregulated and non-productive angiogenesis.^4^ The degree of tumour hypoxia has been shown to be associated with decreased patient survival.^5^ Hypoxia gene expression signatures have demonstrated that breast cancers are hypoxic and particularly ER-negative (ER−), PR-negative (PR−), and TNBC tumours.^6,7^ Hypoxia-inducible factors (HIFs) are transcription factors consisting of alpha (HIF1α and HIF2α) and beta (HIF1β/ARNT) subunits, which act as key regulators of hypoxic biology by regulating the expression of genes involved in pro-tumourigenic and pro-metastatic pathways.^3,8^ Recently, the E3 ligase WSB-1 (WD-40 repeat-containing SOCS Box protein) was identified as a transcriptional target of HIF.^9,10^ WSB-1 is the substrate recognition element of an Elongin Cullin SOCS (ECS box) E3 ubiquitin ligase complex.^11^ It has been proposed that a specific isoform of WSB-1 promoted cell proliferation in pancreatic cancer models^12^ and that WSB-1 was involved in chemoresistance in hepatocellular cancer cells.^10^ WSB-1 expression was recently associated with increased metastatic potential in osteosarcoma and lung adenocarcinoma.^9,13^ Therefore, WSB-1 appears to play an important role in tumour progression and resistance to chemotherapy, but these effects appear to be tumour-type specific, and the precise WSB-1 mechanisms of action remain elusive.

This study is the first to investigate the role of WSB-1 in breast cancer biology and progression, particularly in HR-independent backgrounds. High *WSB-1* expression was associated with decreased distant metastasis-free survival (DMFS) only for HR-negative cases. Interestingly, although WSB-1 was induced in hypoxic conditions, it was not associated with a canonical hypoxic response in breast cancer. WSB-1 knockdown led to decreased cellular invasion of HR-negative cell line models. Importantly, this study is the first to show that WSB-1 knockdown led to decreased angiogenic potential, through modulation of metalloproteinase (MMP) expression and activity and vascular endothelial growth factor (VEGF) secretion. Finally, we showed that knockdown of WSB-1 in this HR-negative model led to decreased metastatic seeding and growth in vivo. This study suggests WSB-1 plays a significant role in breast cancer, particularly in a HR and hypoxic signalling-independent context.

## Materials and methods

### Cell lines and hypoxia treatment

MDA-MB-231, MDA-MB-468, MDA-MB-361, MDA-MB-436, BT474, T47D, and MCF-7 breast cancer cell lines and Human Mammary Epithelial Cells (HMEpC) were purchased from ATCC (US) or ECCAC (UK). Breast cancer lines were grown in DMEM (Corning) and HMEpC in HMEC Growth Medium (Lonza), both supplemented with 10% FBS. All cell lines were routinely tested as negative for mycoplasma. Cells were maintained at 5% CO_2_ and 37 ^o^C. Hypoxia treatments were performed in a H35 Hypoxystation (Don Whitley Scientific) with humidified atmosphere containing 2% O_2_, 5% CO_2_ at 37 ^o^C.

### Absolute quantitative PCR

A standard curve was prepared containing known number of WSB-1 copies, using the pFLAG-CMV2 plasmid containing the WSB1 gene (a kind gift from Prof. Hironobu Asao, Yamagata University, Japan) as a template.^14^ The standard samples were analysed alongside unknown samples using QuantiFAST SYBR Green (Qiagen) in the StepOnePlus™ Real-Time PCR System (Thermo Scientific).

### Quantitative real-time PCR

Transcript levels in cell line samples were monitored by QuantiFAST SYBR Green (Qiagen) using the StepOnePlus™ Real-Time PCR System (Thermo Scientific). Transcript expression levels were normalised to *B2M* (β-2-microglobulin). Primer sequences are available in Supplementary Information.

Transcript levels in TissueScan breast microarray plates (panels I, II, IV) (OriGene) were analysed using TaqMan qPCR SsoAdvanced Universal Probes for *WSB1* (qHsaCIP0050519), *CA9* (qHsaCIP0031395) (BioRad), and *B2M* (4326319E) (Applied Biosystems). Patient information is available in Supplementary Information.

### Breast cancer patient DMFS analysis

Kaplan–Meier curves for DMFS were generated using the KM-plotter tool (http://kmplot.com/analysis).^15^ The analysis used microarray data from 1809 breast cancer patients. Analyses were performed for the following groups: all patients, ER+, ER−, PR+, or PR− patients. Analysis of *WSB1* expression was performed using the mean expression of four *WSB1* Affymetrix probes: 201294_s_at, 201295_s_at, 201296_s_at, and 210561_s_at. Patients were grouped as having high or low *WSB1* expression, and median expression was used as the cut-off.

### Gene expression correlation analysis in cancer datasets

RNA-sequencing datasets (RNA Seq V2 RSEM) for breast invasive carcinoma tumours were downloaded from the TCGA project accessed through cBioportal (http://www.cbioportal.org). These datasets included all patients (*n* = 1110), ER+ patients (*n* = 593) and ER− patients (*n* = 174). To examine *WSB1* expression against hypoxia metagene signature^16^ or the 26-gene hypoxia signature,^17^ raw data for each sequenced gene and *WSB1* were rescaled to set the median equal to 1. Expression values for hypoxia signatures was determined by quantifying the median expression of signature genes. Log_10_ conversion of the hypoxia signatures were plotted against Log_10_ conversion of rescaled data for *WSB1*.

### Cell lysis and Western blotting

Whole cell lysates (WCL) were prepared using UTB (9 M urea, 75 mM Tris–HCl pH 7.5 and 0.15 M β-mercaptoethanol) and immunoblotted as previously.^18^ For protein presence in conditioned media, cells were incubated for 24 h with serum-free media and media was concentrated using Vivaspin 4 ultrafiltration spin column (Sartorius). Antibodies used were anti-WSB-1 (Genetex), anti-HIF1α (BD-Biosciences), anti-MMP1 (R&D Systems), anti-MMP2 (Cell Signaling Technology), and anti-MMP14 (Abcam). Anti-GAPDH (BD-Biosciences) and anti β-actin (Santa Cruz) were used as loading controls. Densitometric analysis of band intensity was performed using ImageJ software (NIH).

### siRNA knockdown

For transient knockdown experiments, cells were transfected using DharmaFECT1 (GE Dharmacon), as per manufacturer’s instructions. siRNA oligos used were: non-targeting siRNA and siGENOME SMARTpool WSB-1 siRNA (GE Dharmacon).

### VEGF ELISA and in vitro angiogenesis branch forming assay

Quantification of VEGF in conditioned media was detected using the VEGF ELISA kit (Invitrogen), as per manufacturer’s instructions.

Human umbilical vein endothelial cells (HUVEC) were grown in complete endothelial cell growth medium (Lonza). Branch forming assay was performed using the In Vitro Angiogenesis Assay Kit (Merck Millipore), as per manufacturers’ instructions. Branching was quantified by scoring the number of branching points in three random fields per replicate per experiment.^19^

### In vitro cellular invasion assays

Cellular invasion assays were performed using Matrigel coated or uncoated transwell chambers (BD Bioscience) as per manufacturer’s instructions and as previously described.^20^ Cells were then counted in three distinct fields per triplicate well, histograms depict average invasion index.

### Gelatine zymography

Conditioned media was processed as described before, and separated through a non-denaturing 10% acrylamide gel containing 1 mg/mL gelatine. Complete media (DMEM supplemented with 10% FBS) was used as a control. Gelatine gel was incubated in renaturing buffer (Novex) 1 h and in 1 × developing buffer (Novex) at 37 °C overnight. Gel was stained in Coomassie-blue solution as previously described.^21^

### Mammosphere formation assay

Mammosphere formation assay was performed as previously described.^22^ In brief, 2500 cells were seeded in wells coated with Matrigel (BD Biosciences, USA) diluted 1:1 in serum-free media. Mammospheres were grown for 10–14 days and imaged. Diameters were determined using ImageJ software (NIH). At least 150 mammospheres were measured per condition.

### Experimental metastasis models

MDA-MB-231 cells were stably transfected with WSB-1 shRNA-targeting or non-targeting shRNA-containing plasmids (OriGene; sequence details in Supplementary Information) using TurboFect as per manufacturer’s instructions (Fisher Scientific). 5 × 10^5^ cells were injected into the tail vein of female CD-1 nude mice aged 5–7 weeks (Charles River) (*n* = 6 per group). All animal work was performed in accordance with the Animals (Scientific Procedures) Act 1986 and the NCRI guidelines for the use of animals in cancer research,^23^ using protocols approved by the local Animal Welfare and Ethical Review Body (AWERB) under Home Office Project License number 60/4549 held by Dr. Cawthorne. Haematoxylin and eosin (H&E) staining of equally distanced paraffin embedded lung sections was performed as previously reported.^18^ Images were acquired using an Eclipse 80i microscope (Nikon), using the Image Pro Premier software (Media Cybernetics). Image analysis was performed using Image J software (NIH).

### Statistical analysis

Statistical significance was determined by Student’s *t*-test (one variable) or two-way ANOVA with Tukey correction (multiple variables). For the tissue cDNA microarray analyses, significance was determined by Kruskal–Wallis test for non-parametric data. Gene expression correlation and statistical significance were determined by calculating Spearman’s rho rank correlation coefficients and two-tailed *P*. Error bars represent mean ± SEM.

## Results

### High WSB1 expression is associated with decreased DMFS in HR-negative breast cancer patients

**Fig. 1:**
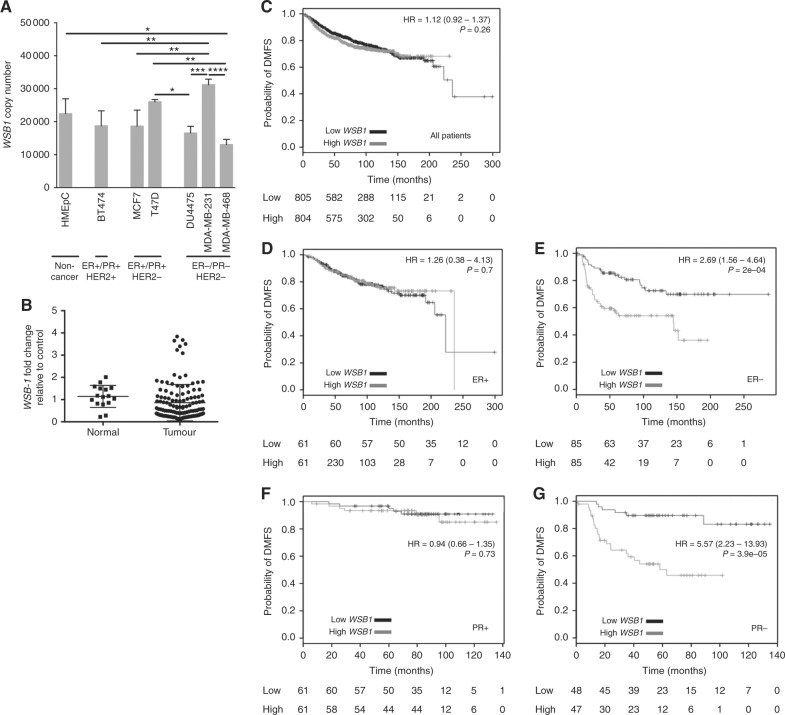
High *WSB1* expression is associated with decreased distant metastasis-free survival (DMFS) in HR-negative breast cancer patients. **a** WSB-1 copy number was measured by absolute qPCR in non-cancer primary breast cell line HMEpC and several breast cancer cell lines. *n* = 3 **b** *WSB1* transcript levels were analysed in tissue cDNA microarrays. Dot plots represent samples arranged as normal (*n* = 16) and tumour (*n* = 128). **c** Kaplan–Meier (KM) plots represent DMFS according to *WSB1* expression in the following cohorts: all patients (*n* = 1609), ER+ (*n* = 122), ER− (*n* = 170), PR+ (*n* = 122), and PR− (*n* = 95). Patients were grouped as having ‘High’ (grey) or ‘Low’ (Black) *WSB1* expression, and median expression used as cut-off. HR hazard ratio *p < 0.05; **p < 0.01; ***p < 0.001; ****p < 0.0001

In order to evaluate the expression of WSB-1 in breast cancer, we analysed the levels of *WSB1* transcript in both a panel of cell lines and patient samples (clinical information noted in Supplementary Table 1). *WSB1* was not shown to be consistently up-regulated or down-regulated between normal mammary epithelial cells and breast cancer cells (Fig. 1a). *WSB1* expression was also not significantly altered in tumours when compared to normal tissue (Fig. 1b). Interestingly, when patient cohorts were clustered by subtype or HR status, *WSB1* expression was significantly lower in the most aggressive tumours, such as HR− and TNBC (Figure S1). Analysis of a larger cohort (TCGA dataset, 1100 patients) also indicated that *WSB1* expression is decreased in breast cancer samples (Figure S2). We then asked whether, within breast cancer patient cohorts, differential WSB-1 expression was associated with differences in survival. For this, we determined the impact of *WSB1* expression on several aspects of patient survival, including OS, relapse free survival (RFS), and DMFS (Fig. 1c–g, S3-S4). Importantly, although *WSB1* expression did not impact DMFS for the whole patient cohort (Fig. 1c), *WSB1* expression levels higher than median were associated with significantly decreased DMFS for ER− and PR− patients (Fig. 1e, g), but not for ER+ or PR+ patients (Fig. 1d, f). There was no impact of *WSB1* expression on OS, but high *WSB1* expression was a poor prognostic indicator for RFS for ER− patients (Figures S3-S4). These data demonstrate that, although *WSB1* expression is not significantly altered between normal and breast tumour tissue, higher *WSB1* expression levels in HR-negative patients are associated with increased likelihood of metastatic disease.

### WSB1 expression is not associated with hypoxic gene expression signatures in patient samples

**Fig. 2:**
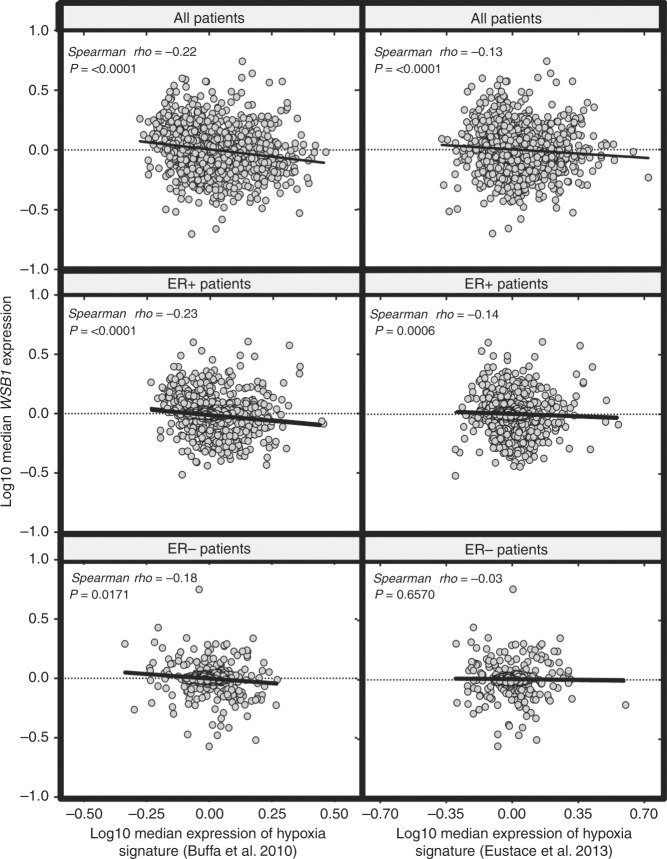
*WSB1* expression is not associated with hypoxic gene expression signatures in patient samples. Correlation expression analysis of *WSB1* gene vs. hypoxia signatures in The Cancer Genome Atlas (TCGA) datasets for breast invasive carcinoma. Log_10_ conversions of *WSB1* median expression against median expression of two independent hypoxia signatures^16, 17^ is shown for all patients (*n* = 1110), ER+ patients (*n* = 593) and ER− patients (*n* = 174). Two-tailed *p* values are shown for Spearman’s rho rank correlation coefficients for each analysis (values inset)

WSB-1 has been previously reported to be a hypoxia-inducible factor in a HIF-dependent manner in osteosarcoma and hepatocellular carcinoma.^9,10^ As hypoxic regions are a common feature in breast cancer and known key drivers of metastatic potential, we were surprised that WSB-1 expression was not elevated in breast cancers compared to normal tissue. However, in breast cancer cell lines, WSB-1 mRNA and protein levels were significantly induced in a HIF1-dependent and HIF2-independent manner (Figure S5). To investigate HIF1-dependent WSB-1 expression in patient samples, we used the TCGA patient cohort dataset to ask whether *WSB1* expression was associated with two well-characterised hypoxia signatures containing well-known HIF targets.^16,17^ Surprisingly, these data showed that WSB1 expression was inversely correlated or not correlated with either hypoxia signatures for all cohorts analysed, which included both ER+ and ER− patients (Fig. 2). This was in contrast with the expression of known HIF target genes involved in breast cancer metastasis not included in the hypoxia signatures (*LOX, LOXL2*, *LOXL4*, *CCL2*, *L1CAM*, *ANGPT1*, and *ANGPT2*), whose expression showed a positive correlation with the signatures (Supplementary Table 2). Taken together, these data indicate that WSB-1 can be induced by HIF1 in hypoxic in breast cancer cell lines, but that this mechanism is unlikely to be the main driver for WSB-1 expression in breast cancers.

### WSB-1 affects the expression of pro-tumourigenic and pro-metastatic factors in a HR-dependent manner

**Fig. 3:**
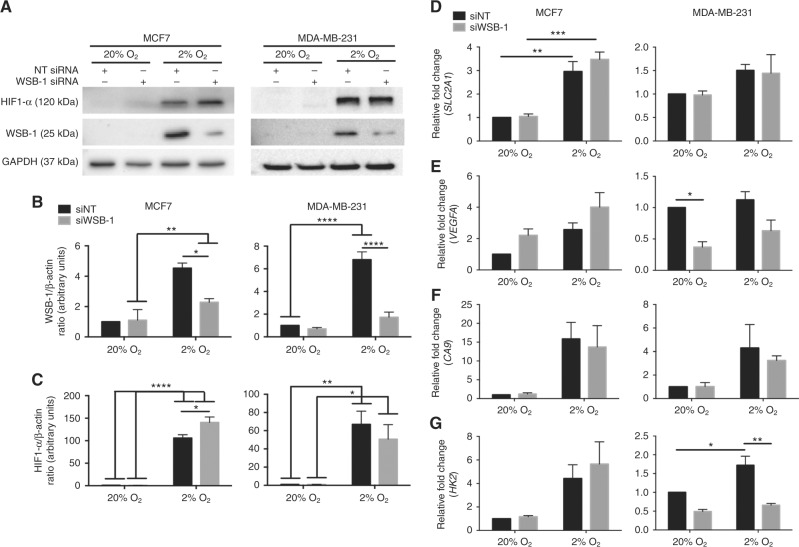
WSB-1 affects the expression of pro-tumourigenic and pro-metastatic factors in a HR-dependent manner MCF7 and MDA-MB-231 cells were transfected with WSB-1 (siWSB-1) or non-targeting siRNA (siNT). Cells were exposed to 20% or 2% O_2_. **a**–**c** WSB-1 and HIF1α protein levels were determined by Western blot. Representative blots (*n* = 3) are shown in **a**, densitometry in **b** and **c**. **d**–**g** Transcript levels of *WSB1* and other HIF-dependent genes were assessed after 24 h exposure to 20% or 2% O_2_. Histograms represent average of *n* = 3 experiments. *p < 0.05; **p < 0.01; ***p  <  0.001;****p < 0.0001

It has been previously reported that WSB-1 could itself regulate the stabilisation of HIF1α via its E3 ligase, VHL. Here, we also observed an impact of WSB-1 knockdown on HIF1α which varied between cell lines (Fig. 3a-c, S6). Specifically, WSB-1 knockdown was associated with a significant upregulation of HIF1α protein expression in MCF7 cells, but not in MDA-MB-231 cells. Surprisingly, this was not reflected on the impact of WSB-1 on the expression of canonical HIF target genes *SLC2A1*, *VEGFA*, *CA9*, and *HK2*. WSB-1 knockdown did not affect the expression of these genes for HR-positive cell lines MCF7 and T47D, suggesting that the increased stabilisation of HIF1 observed (Fig. 3a) was not significant to HIF activity (Fig. 3d–g, S7). Interestingly, in HR-negative cell line MDA-MB-231, WSB-1 knockdown led to a significant decrease in *VEGFA* and *HK2* expression in both normoxia and hypoxia (Fig. 3e, g), with a similar trend observed for MDA-MB-468 HR-negative cells (Figure S7). Finally, we investigated whether there was any link between the expression of *WSB1* and HIF targets *SLC2A1*, *VEGFA*, *CA9*, and *HK2* in breast cancer patients. In contrast to *WSB1* expression, which was not different or decreased between normal tissue and tumour tissue (Fig. 1, S2), the expression of all these genes was increased in the tumour tissue (Figure S1). Furthermore, we evaluated the correlation between *WSB1* expression and *SLC2A1*, *VEGFA*, *CA9*, and *HK2* expression in the TCGA dataset. Whereas there was a significantly positive correlation between *WSB1* and *VEGFA* expression, no significant correlation was observed between the WSB1 and the other genes (Figure S8). These data show that WSB-1 can impact the expression of pro-tumourigenic and pro-metastatic factors, potentially in a HIF-independent manner.

### WSB-1 regulates angiogenic potential in vitro

**Fig. 4:**
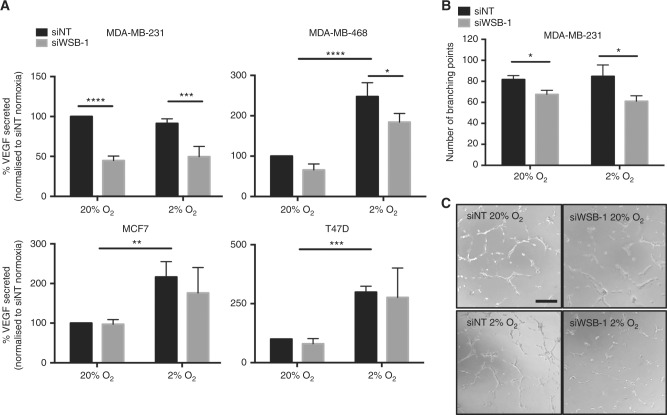
WSB-1 regulates angiogenic potential in vitro. **a** MDA-MB-231, MDA-MB-468, MCF7, and T47D cells were transfected with WSB-1 (siWSB-1) or non-targeting siRNA (siNT), and exposed 24 h to 20% or 2% O_2_. VEGF levels in conditioned media were quantified by ELISA. Histogram represents average of *n* = 3 experiments. **b** Angiogenic potential was assessed using in vitro branch forming assays using conditioned media from MDA-MB-231 cells transfected with siWSB-1 or siNT. Histogram represents average of *n* = 3 experiments. **c** Inset microscopy images are representative of each condition. Scale bar represents 200 μm. *p < 0.05; **p < 0.01; ***p < 0.001; ****p < 0.0001

As we observed an impact of WSB-1 on the expression of the pro-angiogenic factor *VEGFA*, we further investigated whether VEGF secretion was also affected by WSB-1 knockdown in a HR-dependent manner. WSB-1 knockdown led to a decrease in VEGF secretion in HR-negative cell lines MDA-MB-231 and MDA-MB-468, but not in HR-positive lines MCF7 and T47D (Fig. 4a). Importantly, this correlated with an effect on angiogenic potential in vitro. HUVEC cells incubated with conditioned media from MDA-MB-231 cells transfected with WSB-1 siRNA had a significantly decreased endothelial branching ability when compared with conditioned media from cells transfected with non-targeting siRNA (Fig. 4b-c). Together, these data implicate WSB-1 in the angiogenic potential of HR-negative breast cancer cell lines.

### WSB-1 regulates invasiveness of HR-negative cells in vitro

**Fig. 5:**
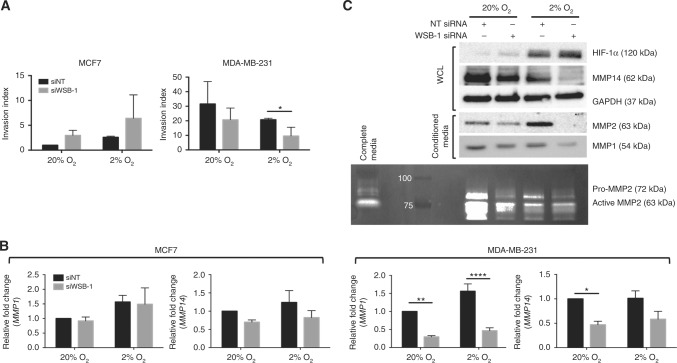
WSB-1 regulates invasiveness of HR-negative cells in vitro MCF7 and MDA-MB-231 cells were transfected with siWSB-1 or siNT. **a** Cells were seeded in control (uncoated) or matrigel-coated transwell inserts and allowed to invade for 18 h at 20% or 2% O_2_. Invasion index = % invasion siWSB-1/% invasion siNT. Histograms represent *n* = 4 experiments. **b** Transcript levels of *MMP1* and *MMP14* were assessed after 24 h exposure to 20% or 2% O_2_. Histograms represent average of *n* = 3 experiments. **c** MDA-MB-231 cells were transfected with either siWSB-1or siNT, and exposed to 20% or 2% O_2_ for 24 h. Whole cell lysates (WCL) or conditioned media samples were analysed by Western blotting for the expression of HIF1α, MMP14, MMP2, and MMP1. Gelatine zymography was used to determine the activity of MMP2 in conditioned media samples. Representative blots and zymograms are shown (*n* = 5). *p < 0.05; **p < 0.01; ***p  <  0.001;****p < 0.0001

We then investigated other mechanisms, besides altered angiogenic potential, by which WSB-1 could impact the likelihood of metastatic spread in a HR-negative context. WSB-1 knockdown led to decreased in vitro invasive potential for MDA-MB-231 cells, but not MCF7 cells, in hypoxia (Fig. 5a). This was not associated with EMT markers expression changes (Figure S9). The impact of WSB-1 knockdown on the expression and activity of MMPs, key for the metastatic process, was also evaluated. WSB-1 knockdown was associated with decreased expression of MMP1 and MMP14 at both transcript and protein level, but again only for MDA-MB-231 HR-negative cells (Fig. 5b, c). The levels of the secreted active form of MMP2, regulated by MMP14, were also decreased by WSB-1 knockdown, with concomitant decrease in MMP2 gelatinase activity (Fig. 5c). These data indicate that WSB-1 regulates the expression and activity of key enzymes involved in increased invasiveness and extracellular matrix (ECM) remodelling.

### WSB-1 promotes metastatic potential in vivo

**Fig. 6:**
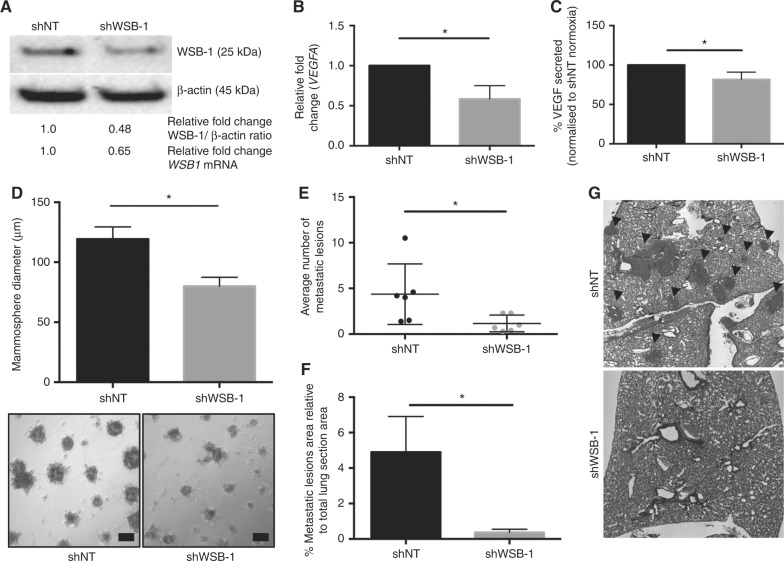
WSB-1 promotes metastatic potential in vivo MDA-MB-231 cells were stably transfected with WSB-1 (shRNA WSB-1) or non-targeting shRNA (shRNA NT) constructs. The WSB-1 shRNA construct #1 was selected, as it led to the most efficient WSB-1 knockdown (Supplementary Figure 10). **a** WSB-1 protein levels were assessed by Western blotting. **b** and **c** Histograms represent *VEGFA* mRNA expression and VEGF secretion as previously described, average of *n* = 3 experiments. **d** Mammospheres were established from shNT and shWSB-1 MDA-MB-231 cells. Histogram represents the average mammosphere diameter per condition per individual experiment. Representative images are shown (*n* = 4). **e**–**g** 5 × 10^5^ cells were injected into the tail vein of female CD-1 nude mice (*n* = 6 per group). After 15 weeks, mice were sacrificed and lung tissue was collected and fixed. For each animal, the right lung was sectioned into 10 µm sections and a total of 10 equally distanced sections were H&E stained, imaged (10× objective), and scored for presence of metastatic foci. The metastatic lesions were also measured. The total number of metastatic foci per lung per group is represented in the scatter plot (**e**), and the histogram represents the average area occupied by metastatic lesions in relation to total lung area (**f**). Representative example of a section of each group is shown in (**g**). Arrowheads indicate the location of metastatic lesions. *p < 0.05

In order to validate our in vitro observation in vivo, we generated WSB-1 shRNA (shWSB-1) cell lines and matching non-targeted controls (shNT) in MDA-MB-231 cells (Fig. 6, S10A). shWSB-1 cells had a lower expression of VEGF both at transcript and protein levels (Fig. 6b and c), similarly to WSB-1 siRNA treated cells (Fig. 4). Interestingly, although there was no difference in the growth rates or cell cycle profiles of the two cell lines when grown in 2D (Figure S10B-C), shWSB-1 cells originated smaller mammospheres in Matrigel when compared with shNT cells (Fig. 6d).

MDA-MB-231 shNT or shWSB-1 cells were then injected into the tail veins of two groups of mice, and metastatic burden in the lungs was evaluated ex vivo after 12 weeks. Importantly, we observed a significantly lower metastatic burden (lesions/lung) for the cohort of animals injected with WSB-1 shRNA cells, when compared to the shRNA NT control cells (Fig. 6e–g) in this experimental metastasis model. This was also reflected in the size of metastatic lesions present. These observations further validate the pro-metastatic phenotype observed in vitro, and indicate that WSB-1 is necessary for the metastatic seeding and growth of HR− breast cancer cells in vivo.

## Discussion

In this study, we have shown that high expression of WSB-1 in breast cancer is a marker for increased metastatic disease in HR-negative breast cancer patients. Downregulation of WSB-1 expression led to decreased angiogenic and invasive potential of HR-negative breast cancer cell lines in vitro and metastatic seeding and growth in vivo (Figure S11).

The present study indicates that increased WSB-1 expression is associated with an increased metastatic propensity in ER− and PR− breast cancer patients. This is in accordance with Cao and colleagues, who demonstrated that higher WSB-1 levels in metastatic osteosarcoma tumours were associated with decreased DMSF, indicating low WSB-1 expression might have a good prognostic value for metastasis-free survival.^9^ Kim and colleagues investigated WSB-1 expression levels in several cancer types (lung adenocarcinoma, melanoma, prostate cancer, and bladder cancer) and found that WSB-1 level was elevated in metastatic tissues when compared to primary tumours, and that this effect was mediated by a regulation of HIF1 function.^13^ Our study is the first to show a link between WSB-1-associated metastatic phenotypes and HR status, in particular linked with angiogenesis promotion. VEGF and other pro-angiogenic factors are known key players during the metastatic cascade, through increased vascularisation and vasculature permeability.^24^ Intravasation and extravasation are reliant on ECM-remodelling, as well as increased vascular permeability. MMP levels can indicate increased invasiveness of cancer cells and reveal an aggressive tumour.^25^ Specifically, MMP1 and MMP14 increased expression in breast cancer has been correlated with poor prognosis.^26,27^ In our study, WSB-1 knockdown led to a decrease in MMP expression and activity. Interestingly, Kim and colleagues found that tumour metastasis with high WSB-1 expression presented increased expression of MMP2 and MMP9 levels in patients with lung cancer.^13^

We propose that, for HR-negative breast cancer patients, WSB-1 is a key regulator of several molecular pathways central to metastatic seeding and growth of HR-negative breast cancer, including remodelling of the ECM by MMPs and increased angiogenic sprouting via MMPs and VEGF. However, the mechanism behind the impact of WSB-1 function in this context remains unclear.

WSB-1 has been previously reported to be upregulated at the gene and protein level in tumour vs. normal tissues for cancer types, such as osteosarcoma and lung adenocarcinoma.^9,13^ However, in our study the trends observed indicated a decrease in *WSB1* expression in patient samples, and particularly in the more aggressive tumour types. These differences could be due to our analysis focusing on *WSB-1* transcript levels, whereas previous studies investigated WSB-1 protein expression, indicating other post-transcriptional regulatory processes may be involved. It could also be associated with the nature of the tissue samples analysed that would be comprised of many different cell types, which could have masked expression pattern changes.

WSB-1 was previously identified as a HIF1 target gene in osteosarcoma and hepatocellular carcinoma.^9,10^ We also observed that WSB-1 was upregulated in hypoxic conditions in vitro in a HIF1-dependent manner in breast cancer cell lines. However, surprisingly, in patient samples *WSB1* expression was inversely correlated with that of two well-characterised hypoxia gene expression signatures, the majority of which are HIF-targets.^16,17^ This is even more surprising considering other HIF-target genes involved in breast cancer metastasis correlated with the hypoxia signatures. These data indicate that the hypoxic microenvironment is not a key contributor for differences in *WSB1* expression in patient samples. It is plausible that WSB-1 regulation by HIF has a role in earlier stages of tumour development, which are not reflected in the later stage patient samples analysed. These data have implications for the link between WSB-1 expression and hypoxia and HIF, and the usefulness of WSB-1 as a hypoxia biomarker.

It has been previously reported that increased WSB-1 activity was associated with altered HIF stabilisation and function.^13^ We observed a similar effect of WSB-1 knockdown on HIF stabilisation in HR-positive, but not in a HR-negative context. Interestingly, this is mirrored to a degree in impact of WSB-1 on the expression of canonical HIF targets, such as *SLC2A1*, *VEGFA*, *CA9*, and *HK2*. In the HR-positive cell line MCF7, WSB-1 knockdown did not significantly alter gene expression (with a non-significant trend for increased expression for *SLC2A1*, *VEGFA*, and *HK2*), which would mirror an increase in HIF levels. In the HR-negative cell line MDA-MB-231, WSB-1 knockdown was associated with significantly decreased expression of *VEGFA* and *HK2*. Furthermore, *WSB1* expression in patient samples did not correlate with several HIF targets including *HK2*, *SLC2A1*, and *CA9* in breast cancer patient samples, unlike that shown in other tumour types.^9,13^ This further indicates that the role of WSB-1 in breast cancer HR-negative cells might not be fully dependent on HIF activity, and that other transcription factors are potentially involved. It is also plausible that WSB-1 could regulate the expression of only a selected number of HIF target genes, rather than all HIF-dependent expression, as it is the case in breast cancer, respectively, for co-factors p300 and DEK for GLUT-1 (*SLC2A1*) and VEGF (*VEGFA*) expression.^28,29^

Breast cancer is the second most represented cancer type worldwide and metastatic (or secondary) breast cancer is responsible for the majority of patient deaths. Improvement of diagnosis and early detection of metastatic propensity, and the identification of relevant molecular players continue to be areas of unmet need. We have shown a novel role for WSB-1 in promotion of metastasis seeding and growth in breast cancer, suggesting that WSB-1 regulates key pathways for the establishment of secondary breast cancer in HR-negative patients, an area identified as of key importance in the field.^30^ Further clarification of the regulatory pathways downstream of WSB-1 will help to further elucidate the biology of these aggressive and invasive breast cancers, and potentially uncover novel potential therapeutic targets.

## Electronic supplementary material

Supplementary materials and methods

Supplementary Table 1

Supplementary Table 2

S1 - Supplementary Figure 1

S2 - Supplementary Figure 2

S3 - Supplementary Figure 3

S4 - Supplementary Figure 4

S5 - Supplementary Figure 5

S6 - Supplementary Figure 6

S7 - Supplementary Figure 7

S8 - Supplementary Figure 8

S9 - Supplementary Figure 9

S10 - Supplementary Figure 10

S11 - Supplementary Figure 11
