# Supplementary material for: WSB-1 regulates the metastatic potential of hormone receptor negative breast cancer
Source: Br J Cancer. 2018 Mar 15;118(9):1229–37. doi: 10.1038/s41416-018-0056-3 (PMC5943535; doi:10.1038/s41416-018-0056-3)
Supplement: Supplementary file 1 — Supplementary materials and methods [file 41416_2018_56_MOESM1_ESM.docx]

**Supplementary Materials and Methods**

**qPCR primer assays and sequences**

Transcript levels of various genes was assessed using the following primer assays (Qiagen) or primer sequences: *B2M* (QT00088935, Qiagen); *WSB1* (QT01018248, Qiagen); *SLC2A1* (QT00068957, Qiagen); *MMP1* (QT00014581); *VEGF* (F:CTACCTCCACCATGCCAAGT - R: CTCGATTGGATGGCAGTAGC); *HK2* (F: TGCCACCAGACTAAACTAGACG - R: TGAATCCCTTGGTCCATGAGA); *CA9* (F: GGAAGGCTCAGAGACTCA - R: CTTAGCACTCAGCATCAC); *MMP14* (F: CCCCGAAGCCTGGCTACA R: GCATCAGCTTTGCCTGTTACT).

**Breast cancer patient overall survival and relapse free survival analysis**

Kaplan-Meier curves for overall survival (OS), and Relapse-Free Survival (RFS) were generated using the KM-plotter online tool. The analysis used microarray data for 1117 (OS), and 3557 (RFS) breast cancer patient cohorts. Analyses were performed for the following group in each cohort: all patients ER+, ER-, PR+, or PR- patients. Analysis of *WSB1* expression was performed using the mean expression of four *WSB1* Affymetrix probes (201294_s_at, 201295_s_at, 201296_s_at, 210561_s_at). Patients were grouped as having high or low *WSB-1* expression, and median expression used as cut-off.

**siRNA knockdown**

For transient knockdown experiments, cells were transfected using DharmaFECT1 (GE Dharmacon), as described previously. Supplementary siRNA oligos used were: HIF-1α (CUGAUGACCAGCAACUUGA dT); HIF-2α (CAGCAUCUUUGAUAGCAGU dT); HIF-1β (GGUCAGCAGUCUUCCAUGA dT).

**Antibodies used in Supplementary Data**

Antibodies used were anti-WSB-1 (Genetex), anti-HIF1α and anti-GAPDH (BD-Biosciences), anti-ZO1, anti-E-Cadherin, anti-Vimentin, anti-HIF-2α, and anti-HIF-1β (Cell Signaling Technology).

**shRNA constructs details**

shRNA constructs were purchased from Origene. shRNA NT (catalogue number TR30012) is a non-effective 29-mer scrambled shRNA cassette in pRS vector. shRNAWSB1 constructs (catalogue number TR317081) are 4 unique 29mer shRNA constructs in untagged pRS vector.

**Propidium iodide staining for cell cycle profile analysis**

Cells were harvested and the cell suspension fixed in 70% Ethanol in 1X PBS. Prior to staining, cells were washed in 1X PBS, and then incubated in 1X PBS with 10 μg/ml of propidium iodide (Sigma, UK) and 100 μg/ml RNAse Sigma, UK). fluorescence-activated cell sorting (FACS) analysis was performed on a FACS Calibur analyser (BD Biosciences), and Data was analysed using FCSalyzer software version 0.9.11-alpha (https://sourceforge.net/projects/fcsalyzer).
