# Supplementary material for: WSB-1 regulates the metastatic potential of hormone receptor negative breast cancer
Source: Br J Cancer. 2018 Mar 15;118(9):1229–37. doi: 10.1038/s41416-018-0056-3 (PMC5943535; doi:10.1038/s41416-018-0056-3)
Supplement: Supplementary file 2 — Supplementary Table 1 [file 41416_2018_56_MOESM2_ESM.docx]

**Supplementary Table 1 - Summary of the clinical features of the breast tumour samples in the TissueScan arrays analysed in this study**

| **Patients characteristics** | | | **Number of patients (n=128)** | | |
| --- | --- | --- | --- | --- | --- |
|  |  |  |  |  |  |
| Age ± standard deviation (years) | | | 56.3 ± 13.1 |  |  |
|  |  |  |  |  |  |
| Tumour Stage | |  |  |  |  |
| I |  |  | 23 |  |  |
| IIA |  |  | 36 |  |  |
| IIB |  |  | 22 |  |  |
| IIIA |  |  | 23 |  |  |
| IIIB |  |  | 6 |  |  |
| IIIC |  |  | 13 |  |  |
| IV |  |  | 5 |  |  |
|  |  |  |  |  |  |
| Tumour Subtypes | |  |  |  |  |
| Luminal A |  |  | 50 |  |  |
| Luminal B |  |  | 12 |  |  |
| HER2 type |  |  | 7 |  |  |
| TNBC |  |  | 25 |  |  |
| Undefined |  |  | 50 |  |  |
|  |  |  |  |  |  |
| Hormone Receptor expression | | |  |  |  |
| ER+ |  |  | 57 |  |  |
| ER- |  |  | 45 |  |  |
| PR+ |  |  | 55 |  |  |
| PR- |  |  | 40 |  |  |
| HER2+ |  |  | 20 |  |  |
| HER2- |  |  | 70 |  |  |
|  |  |  |  |  |  |
| ER: oestrogen receptor, PR: progesterone receptor, HER2: human epidermal growth factor receptor, TNBC: triple negative breast cancer, +: positive, -: negative. | | | | | |
