# Supplementary material for: WSB-1 regulates the metastatic potential of hormone receptor negative breast cancer
Source: Br J Cancer. 2018 Mar 15;118(9):1229–37. doi: 10.1038/s41416-018-0056-3 (PMC5943535; doi:10.1038/s41416-018-0056-3)
Supplement: Supplementary file 3 — Supplementary Table 2 [file 41416_2018_56_MOESM3_ESM.docx]

**Supplementary Table 2 – Correlation expression analysis of known HIF gene targets involved in metastasis in breast cancer vs hypoxia signatures.**

Log10 conversions of HIF gene targets involved in breast cancer metastasis (*LOX, LOXL2*, *LOXL4*, *CCL2*, *L1CAM*, *ANGPT1*, and *ANGPT2*) median expression against two independent hypoxia signatures (Buffa *et al*, 2010; Eustace *et al*, 2013) median expression was analysed for breast invasive carcinoma for all patients (n=1110) from the The Cancer Genome Atlas (TCGA) datasets for breast invasive carcinoma. Spearman’s rho rank correlation coefficients (and corresponding two-tailed *P* values) are noted for each analysis. Correlation values for *VEGFA* and *WSB1* are shown for comparison.

| **Gene name** | **Buffa *et al* 2010** | | **Eustace *et al* 2013** | |
| --- | --- | --- | --- | --- |
|  | Spearman rho | *P* value | Spearman rho | *P* value |
| *LOX* | 0.03408 | 0.2588 | **0.1133** | **0.0002** |
| *LOXL2* | **0.1874** | **< 0.0001** | **0.1897** | **< 0.0001** |
| *LOXL4* | **0.08653** | **0.0041** | **0.1236** | **< 0.0001** |
| *CCL2* | **0.2377** | **< 0.0001** | **0.115** | **0.0001** |
| *L1CAM* | **0.3373** | **< 0.0001** | **0.1353** | **< 0.0001** |
| *ANGPT1* | 0.004561 | 0.8799 | **0.071** | **0.0188** |
| *ANGPT2* | 0.04705 | 0.1188 | **0.09567** | **0.0015** |
|  |  |  |  |  |
| *WSB1* | **-0.2179** | **< 0.0001** | **-0.1311** | **< 0.0001** |
|  |  |  |  |  |
| *VEGFA* | **0.1105** | **0.0002** | **0.3326** | **< 0.0001** |
