# Supplementary material for: WSB-1 regulates the metastatic potential of hormone receptor negative breast cancer
Source: Br J Cancer. 2018 Mar 15;118(9):1229–37. doi: 10.1038/s41416-018-0056-3 (PMC5943535; doi:10.1038/s41416-018-0056-3)
Supplement: Supplementary file 4 — S1 - Supplementary Figure 1 [file 41416_2018_56_MOESM4_ESM.docx]

**Supplementary Figure 1 – *WSB1* expression in breast cancer patient samples**

**grouped by subtype vs. normal tissue and hormone receptor subtype**

*WSB1* transcript levels were analysed in tissue cDNA microarrays (TissueScan panels I, II, IV) as previously described. (A) Dot plots represent samples arranged according to breast cancer subtype: Normal (non-cancer, n=16), Luminal A (n=50), Luminal B (n=12), HER2 + (HER2 type, n=7) and TNBC (n=25). (B - C) Dot plots represent samples arranged according to hormone receptor status: Normal (non-cancer, n=16), ER+ (n=57), ER- (n=44), PR+ (n=55), PR- (n=40). * *p*<0.05; ** *p*<0.01
