# Supplementary material for: WSB-1 regulates the metastatic potential of hormone receptor negative breast cancer
Source: Br J Cancer. 2018 Mar 15;118(9):1229–37. doi: 10.1038/s41416-018-0056-3 (PMC5943535; doi:10.1038/s41416-018-0056-3)
Supplement: Supplementary file 5 — S2 - Supplementary Figure 2 [file 41416_2018_56_MOESM5_ESM.docx]

**Supplementary Figure 2 – Expression of *WSB1* and canonical HIF targets in normal tissue vs breast carcinoma (TCGA dataset)**

Analysis of *WSB1*, *SLC2A1*, V*EGFA*, and H*K2* expression in normal vs breast carcinoma tissue in the TCGA dataset was performed using the Oncomine database ([www.oncomine.org](http://www.oncomine.org/)). Normal breast tissue (n=61) compared to invasive ductal breast carcinoma (n=389), invasive lobular breast carcinoma (n=36), and mucinous breast carcinoma (n=4).
