# Supplementary material for: WSB-1 regulates the metastatic potential of hormone receptor negative breast cancer
Source: Br J Cancer. 2018 Mar 15;118(9):1229–37. doi: 10.1038/s41416-018-0056-3 (PMC5943535; doi:10.1038/s41416-018-0056-3)
Supplement: Supplementary file 6 — S3 - Supplementary Figure 3 [file 41416_2018_56_MOESM6_ESM.docx]

**Supplementary Figure 3 – Altered *WSB1* expression is not associated with overall survival in breast cancer patients**

KM plots represent overall survival (OS) according to *WSB1* expression level in the following cohorts: all patients, ER+, ER-
