# Supplementary material for: WSB-1 regulates the metastatic potential of hormone receptor negative breast cancer
Source: Br J Cancer. 2018 Mar 15;118(9):1229–37. doi: 10.1038/s41416-018-0056-3 (PMC5943535; doi:10.1038/s41416-018-0056-3)
Supplement: Supplementary file 7 — S4 - Supplementary Figure 4 [file 41416_2018_56_MOESM7_ESM.docx]

**Supplementary Figure 4 – High *WSB1* expression is associated with decreased relapse free survival in ER- breast cancer patients**

KM plots represent relapse free survival (RFS) according to *WSB1* expression level in the following cohorts: all patients, ER+, ER-, PR+, PR-.
