# Supplementary material for: WSB-1 regulates the metastatic potential of hormone receptor negative breast cancer
Source: Br J Cancer. 2018 Mar 15;118(9):1229–37. doi: 10.1038/s41416-018-0056-3 (PMC5943535; doi:10.1038/s41416-018-0056-3)
Supplement: Supplementary file 8 — S5 - Supplementary Figure 5 [file 41416_2018_56_MOESM8_ESM.docx]

**Supplementary Figure 5 – WSB-1 expression is induced in hypoxic conditions in breast cancer cell lines in a HIF1α-dependent but HIF2α-independent manner**

(A) *WSB1* transcript levels were assessed after 24h exposure to 20% or 2% O_2_ for a panel of breast cancer cell lines. Histogram represents average of n=3 experiments. (B) MCF7 and MDA-MB-231 cells were exposed to 2% O_2_ for the periods indicated and WSB-1 and HIF1α protein levels were determined by Western blot. Representative blots are shown (n=3), densitometry shown below. (C) MCF7 cells were transfected with HIF1α (siHIF-1α/ si1α), HIF2α (siHIF-2α/si2α), HIF1β (siHIF-1β/si1β), or non-targeting siRNA (siNT). Cells were exposed to 20% or 2% O_2_ for 24h and *WSB1* transcript levels was assessed by qPCR. Histogram represents average of n=3 experiments. Knockdown efficiency for HIF isoforms is shown in (D), representative of n=3 experiments. * *p*<0.05
