# Supplementary material for: WSB-1 regulates the metastatic potential of hormone receptor negative breast cancer
Source: Br J Cancer. 2018 Mar 15;118(9):1229–37. doi: 10.1038/s41416-018-0056-3 (PMC5943535; doi:10.1038/s41416-018-0056-3)
Supplement: Supplementary file 9 — S6 - Supplementary Figure 6 [file 41416_2018_56_MOESM9_ESM.docx]

**Supplementary Figure 6 – *WSB1* knockdown efficiency (mRNA level)**

MCF7 and MDA-MB-231 cells were transfected with WSB-1 (siWSB-1) or non-targeting siRNA (siNT). Transcript levels of *WSB1* were assessed after 24h exposure to 20% or 2% O_2_. Histograms represent average of n=3 experiments. Western blots represent the efficiency of knockdown at protein level. *** *p*<0.001; **** *p*<0.0001
