# Supplementary material for: WSB-1 regulates the metastatic potential of hormone receptor negative breast cancer
Source: Br J Cancer. 2018 Mar 15;118(9):1229–37. doi: 10.1038/s41416-018-0056-3 (PMC5943535; doi:10.1038/s41416-018-0056-3)
Supplement: Supplementary file 10 — S7 - Supplementary Figure 7 [file 41416_2018_56_MOESM10_ESM.docx]

**Supplementary Figure 7 – WSB-1 expression is not associated with HIF target gene expression in breast cancer**

T47D and MDA-MB-468 cells were transfected with WSB-1 (siWSB-1) or non-targeting siRNA (siNT). Transcript levels of WSB1 and other HIF-dependent genes were assessed after 24h exposure to 20% or 2% O2. Histograms represent average of n=3 experiments.

* *p*<0.05; ** *p*<0.01
