# Supplementary material for: WSB-1 regulates the metastatic potential of hormone receptor negative breast cancer
Source: Br J Cancer. 2018 Mar 15;118(9):1229–37. doi: 10.1038/s41416-018-0056-3 (PMC5943535; doi:10.1038/s41416-018-0056-3)
Supplement: Supplementary file 11 — S8 - Supplementary Figure 8 [file 41416_2018_56_MOESM11_ESM.docx]

**Supplementary Figure 8 – Correlation of *WSB1* expression with canonical HIF target genes in patient samples**

Expression analysis was performed using The Cancer Genome Atlas (TCGA) datasets for breast invasive carcinoma. Log10 conversions of *WSB1* median expression against canonical HIF target genes *SLC2A1*, *VEGFA*, *CA9*, and *HK2* median expression is shown for breast invasive carcinoma for all patients (n=1110). Two-tailed *P* values are shown for Spearman’s rho rank correlation coefficients for each analysis (values inset in corresponding plots).
