# Supplementary material for: WSB-1 regulates the metastatic potential of hormone receptor negative breast cancer
Source: Br J Cancer. 2018 Mar 15;118(9):1229–37. doi: 10.1038/s41416-018-0056-3 (PMC5943535; doi:10.1038/s41416-018-0056-3)
Supplement: Supplementary file 12 — S9 - Supplementary Figure 9 [file 41416_2018_56_MOESM12_ESM.docx]

**Supplementary Figure 9 – Expression of EMT markers in response to WSB-1 knockdown**

MCF7 and MDA-MB-231 cells were transfected with siWSB-1 or siNT. Cells were exposed to 2% O_2_ for the periods indicated and ZO-1, E-Cadherin, Vimentin protein levels were determined by Western blotting. Blots are representative of n=3 experiments (A). Histograms represent average band intensity relative to loading control (β-actin) of n=3 experiments (B). No statistical differences were observed.
