# Supplementary material for: WSB-1 regulates the metastatic potential of hormone receptor negative breast cancer
Source: Br J Cancer. 2018 Mar 15;118(9):1229–37. doi: 10.1038/s41416-018-0056-3 (PMC5943535; doi:10.1038/s41416-018-0056-3)
Supplement: Supplementary file 13 — S10 - Supplementary Figure 10 [file 41416_2018_56_MOESM13_ESM.docx]

**Supplementary Figure 10 – Generation and characterisation of WSB-1 shRNA cell lines**

(A) MDA-MB-231 cells were stably transfected with WSB-1 (shRNA WSB-1) or non-targeting shRNA (shRNA NT) constructs. Four WSB-1 shRNA constructs were used (#1, #2, #3, #4), encoding for different WSB-1 shRNA sequences. Transcript levels of *WSB1* were assessed by qPCR for shRNA NT and all shRNA WSB-1 cell lines. *B2M* was used as a housekeeping gene. Histogram represents the average of n=3 experiments. (B-C) shNT (shRNA NT) and shWSB-1(shRNA WSB-1) MDA-MB-231 cells have no growth or cell cycle phenotypical differences. shNT and shWSB-1 cells were seeded in 24-well plates, and cell numbers were quantified for 7 days (B). Cell cycle profiles were evaluated for shNT and shWSB-1 MDA-MB-231 cell lines (C).
