# Supplementary material for: WSB-1 regulates the metastatic potential of hormone receptor negative breast cancer
Source: Br J Cancer. 2018 Mar 15;118(9):1229–37. doi: 10.1038/s41416-018-0056-3 (PMC5943535; doi:10.1038/s41416-018-0056-3)
Supplement: Supplementary file 14 — S11 - Supplementary Figure 11 [file 41416_2018_56_MOESM14_ESM.docx]

**Supplementary Figure 11 – Diagram representative of the impact of WSB-1 in the metastatic cascade in HR-negative cells.**
